# Temperature Effect on the Growth and Development of *Habrobracon hebetor* Say (Hymenoptera: Braconidae) Reared on *Ephestia elutella* (Hübner) (Lepidoptera: Pyralidae) Larvae

**DOI:** 10.3390/insects15050336

**Published:** 2024-05-07

**Authors:** Yong Huang, Wenjing Liu, Jianhua Lü, Wenkai Wang, Yafei Guo

**Affiliations:** 1 College of Agriculture, Yangtze University, Jingzhou 434000, China; heiyingsiyu@163.com; 2 Henan Collaborative Innovation Center for Grain Storage Security, School of Food and Strategic Reserves, Henan University of Technology, Zhengzhou 450001, China

**Keywords:** *Habrobracon hebetor*, temperature, life history, growth and development

## Abstract

**Simple Summary:**

The present study investigated the effects of different temperatures (15, 20, 25, 30, and 35 °C) on the growth and development of *Habrobracon hebetor* Say (Hymenoptera: Braconidae) on *Ephestia elutella* (Hübner) (Lepidoptera: Pyralidae) larvae. *Habrobracon hebetor* could complete growth and development when *E. elutella* larvae were used as hosts, and the developmental duration decreased with increasing temperature at 15, 20, 25, 30, and 35 °C. Taking into account factors such as the total number of eggs laid, egg hatchability, the number of emerged adults, emergence ratio, percentage of female offspring, innate rate of increase, and net reproductive rate, the optimal temperature for rearing of *H. hebetor* is 30 °C.

**Abstract:**

Augmentative release of parasitoids has been an important component of integrated insect management for stored product protection. Understanding the effect of different temperatures on the growth and development of parasitoids is in favor of mass rearing of parasitoids. *Habrobracon hebetor* Say (Hymenoptera: Braconidae) is a highly cosmopolitan, gregarious ecto-parasitoid of a variety of Lepidopterous larvae. Thus, the growth and development of *H. hebetor* reared on *Ephestia elutella* (Hübner) (Lepidoptera: Pyralidae) larvae were investigated at 15, 20, 25, 30, and 35 °C. *Habrobracon hebetor* could complete growth and development, and the developmental duration decreased with increasing temperature at 15, 20, 25, 30, and 35 °C. The development threshold temperatures of *H. hebetor* eggs, larvae, pupae, and egg-to-adult stages were 13.89, 6.39, 9.24, and 9.29 °C, and the effective accumulated temperatures were 23.33, 46.40, 142.68, and 240.31 °C·d, respectively. The total number of eggs laid by *H. hebetor*, the hatching rate of *H. hebetor* eggs, and the percentage of female offspring reached the maximum of 192.39, 83.89%, and 74.04% at 30 °C, respectively. There was no significant difference in pupal survival rate in the temperature range of 15 °C to 35 °C. At 30 °C, the pre-oviposition duration of *H. hebetor* was the shortest (0.87 d). Therefore, the optimal rearing temperature of *H. hebetor* was 30 °C. The present results are useful for the large-scale rearing of *H. hebetor* using *E. elutella* larvae as hosts and effectively implementing the biological control of stored-product insects.

## 1. Introduction

Biological control by inundated releases of the parasitoids is a promising strategy for combating stored-product insects due to increasing negative effects resulting from a long-term use of conventional insecticides, such as insecticide residues in food products, environmental contamination, and so forth. *Habrobracon hebetor* Say (Hymenoptera: Braconidae) is a highly cosmopolitan, gregarious ecto-parasitoid of a variety of Lepidopterous larvae occurring in grain storage spaces, such as *Plodia interpunctella* (Hübner), *Ephestia elutella* (Hübner), *Anagasta kuehniella* Zeller, and *Corcyra cephalonica* (Stainton), which frequently infest stored cereals, beans, oilseeds, rice, wheat, dried fruits, and medicinal materials [1,2,3].

The large-scale rearing of parasitoids is crucial for the successful implementation of biological control. Host species significantly affect the growth and development of parasitoids [4]. Therefore, screening the most suitable host species for *H. hebetor* mass rearing is imperative to effectively control Lepidopterous larvae occurring in grain storage spaces. Dabhi et al. [5] evaluated the biology of *H. hebetor* on seven Lepidopteran larvae, *Sitotroga cerealella* (Olivier), *C. cephalonica* (Stainton), *Galleria mellonella* Linn, and *Earias vittella* (Fab.) at ambient temperatures. *Corcyra cephalonica* was the best host for *H. hebetor* mass rearing among the tested Lepidopteran larvae.

*Ephestia elutella* is the most destructive Lepidopterous pest during tobacco storage. *Ephestia elutella* larvae frequently cause serious loss by feeding stored tobacco and its products [6]. Meanwhile, *E. elutella* larvae feeding may result in increasing moisture and temperature which is in favor of mold development in stored tobacco and its products [7]. *Habrobracon hebetor* can effectively attack *E. elutella* larvae. Parasitic rate of *H. hebetor* on *E. elutella* larvae was more than 70% at the peak period of their host larvae occurrence in tobacco storage warehouse in Guizhou, China, from mid-late June to mid-late August [8]. Therefore, *H. hebetor* has great potential for controlling *E. elutella*.

*Habrobracon hebetor* is sensitive to changes in environmental temperature, which is an important factor affecting the growth and development of insects [9]. There have been many studies on the effects of temperature on the growth and development of parasitoids. The developmental duration and adult life span of *Microplitis similis* Lyle (Hymenoptera: Braconidae) decrease with increasing temperature from 18 °C to 33 °C, and the maximum fecundity appears at 27 °C and 30 °C [10]. *Brachymeria lasus* Walker (Hymenoptera: Chalcididae) can parasitize pupae of *E. elutella*. Its developmental period decreased, and its development rate increased with increasing temperature from 16 °C to 32 °C [8,11]. The developmental starting temperatures of *H. hebetor* eggs, larvae, and pupae are 10.12, 13.24, and 10.60 °C, respectively [12]. Egg hatching rate of *Anisopteromalus calandra* (Howard) (Hymenoptera: Pteromalidae) first increased and then decreased with increasing temperature. The highest hatching rate of *A. calandra* reached 88.67% at 25 °C. The developmental duration and adult life span of *A. calandra* decreased with increasing ambient temperature [13]. The longest development duration of *H. hebetor* larvae on *G. mellonella* larvae was 6.60 d at 20 °C [14].

However, the growth and development of *H. hebetor* on *E. elutella* larvae is meagre so far, which is of great significance for the large-scale breeding of *H. hebetor* and its efficient utilization for controlling *E. elutella*. Thus, the present study aims to investigate the effects of different temperatures (15, 20, 25, 30, and 35 °C) on the growth and development of *H. hebetor* on *E. elutella* larvae.

## 2. Materials and Methods

### 2.1. Insects

*Habrobracon hebetor* was obtained from Guangdong Institute of Grain Science (Guangzhou, China) and reared on 5th instar larvae of *E. elutella* in the laboratory at the Institute of Stored Product Insects of Henan University of Technology, Zhengzhou, China. *E. elutella* was maintained on sterilized artificial diet (wheat bran/glycerin/honey/distilled water (87:5:5:3)). To obtain 5th instar larvae of *E. elutella* for the following experiments, *E. elutella* adults were allowed to oviposit on the artificial diet for 3 d, and then, the artificial diet was kept in an incubation chamber for 35 d at 30 ± 2 °C, 75 ± 5% relative humidity (RH), and a photoperiod of 12:12 h (L:D).

### 2.2. Temperature-Dependent Longevity and Fecundity of Adult H. hebetor

Groups of 15 *E. elutella* 5th instar larvae were placed in a plastic box (a bottom diameter of 5.5 cm, a top diameter of 7.3 cm, and a height of 3.5 cm, containing about 3 g sterilized artificial diet). The boxes were, respectively, maintained at 15, 20, 25, 30, and 35 °C in incubators, and one pair (male/female) of *H. hebetor* (1–2-day-old) adults was introduced into each box. After 24 h, the *H. hebetor* adults were transferred into a new plastic box containing another group of 15 5th instar larvae of *E. elutella*. The number of eggs laid by the *H. hebetor* on the previous group of 15 *E. elutella* 5th instar larvae was checked under a microscope. Then, the previous group of 15 *E. elutella* 5th instar larvae with *H. hebetor* eggs were placed in the original plastic box at 30 ± 2 °C, 75 ± 5% relative humidity (RH), and a photoperiod of 12:12 h (L:D). This operation process was repeated every 24 h until the death of the *H. hebetor*. The total number of eggs, the emergence rate of *H. hebetor* in each plastic box, the percentage of female offspring, and the life span of the offspring males and females were recorded. Ten replicates were conducted.

### 2.3. Temperature-Dependent Survival and Development Time of H. hebetor

Each four pairs of *H. hebetor* adults (female/male = 1:1, 1–2-day-old) were released into one plastic box containing 25 5th instar larvae of *E. elutella*. The *H. hebetor* adults were removed after 24 h, and 30 eggs of *H. hebetor* on *E. elutella* larvae were obtained under a microscope (there were 3–6 eggs of *H. hebetor* on one *E. elutella* larva). Then, the plastic boxes were maintained at 15, 20, 25, 30, and 35 °C, and 70 ± 5% RH, respectively. The development of *H. hebetor* eggs was observed until adult emergence at 6:00, 14:00, and 22:00 every day. The egg hatchability, egg developmental duration, larval development duration and survival rate, pupal development duration, emergence rate, net productive rate, innate rate of increase, and population doubling time of *H. hebetor* were calculated. Fifteen replicates were conducted. A total of 2250 eggs (30 eggs per treatment × 5 temperatures × 15 replicates = 2250 eggs) were used for the experiment.

### 2.4. Data Analysis

Temperature and sex (male and female) were used as independent variables, and the adult longevity was used as a response variable for two-way analysis of variance (ANOVA). Temperature was used as an independent variable, and the fecundity was used as a response variable for one-way analysis of variance (ANOVA). Temperature and life stage were used as independent variables, and egg hatchability, survival rate of larvae and pupae of *H. hebetor*, and developmental duration were used as response variables for two-way analysis of variance (ANOVA). The adult longevity, fecundity, and developmental duration of *H. hebetor* were transformed to log_10_(*x* + 1) scale, and survival rate and egg hatchability were transformed to arcsine square-root values before being subjected to ANOVA analysis for homogeneity of variances with Tukey’s test for mean separation at *p* < 0.05. The equations for the linear regression between temperature (*T*) and developmental rate (*V*) for each life stage of *H. hebetor* at different temperatures were defined. SPSS23.0 was used for data analysis.

The developmental duration (*N*) of eggs at each temperature was converted into the developmental rate (*V* = 1/*N*), and the developmental threshold temperature (*C*) and effective accumulated temperature (*K*) were calculated using the following equations.

(1) $$T^{\prime }=C+KV$$

(2) $$C=\frac{\sum V^{2}\cdot \sum T-\sum V\sum VT}{n\sum V^{2}-{\left(\sum V\right)}^{2}}$$

(3) $$K=\frac{n\sum VT-\sum V\cdot \sum T}{n\sum V^{2}-{\left(\sum V\right)}^{2}}$$

(4) $$s_{c}=\sqrt{\frac{\sum {\left(T-T^{\prime }\right)}^{2}}{n-2}\left[\frac{1}{n}+\frac{{\bar{V}}^{2}}{\sum {\left(V-\bar{V}\right)}^{2}}\right]}$$

(5) $$s_{k}=\sqrt{\frac{\sum {\left(T-T^{\prime }\right)}^{2}}{\left(n-2\right)\cdot \sum {\left(V-\bar{V}\right)}^{2}}}$$

In Equations (1)–(5), *C* represents the developmental threshold temperature, *K* is the effective accumulated temperature, n is the number of insects, *T′* is the theoretical value of temperature, *T* is the test temperature, and *S_C_* and *S_K_* are the standard errors of the developmental threshold temperatures (*C*) and effective accumulated temperatures (*K*).

A predictive equation of the development time of various developmental stages of *H. hebetor* was established as follows:

(6) $$N=\frac{K\pm S_{k}}{T-\left(C\pm S_{c}\right)}$$

The exact value of intrinsic increase rate (*r_m_*) was calculated using stepwise approximation method.

(7) $$\sum_{x=o}^{\infty }e^{{-r}_{m}x}L_{x}^{f}m_{x}^{f}=1$$

In Equation (7), *x* represents the median age, where $L_{x}^{f}$ is the mean survival probability from age *x* to *x* + 1, and $m_{x}^{f}\;$ is the number of female offspring produced per female in the interval *x*.

## 3. Results

### 3.1. Temperature-Dependent Longevity of H. hebetor Adults

Temperature significantly affected the longevity of *H. hebetor* females (*F* = 928.14; *df* = 4.45; *p* < 0.01), which decreased with increasing temperature (Table 1). The longevity of females were 51.41 d and 6.69 d at 15 °C and 35 °C, respectively. The longevity of males was the shortest at 35 °C and the longest at 15 °C. The longevity of *H. hebetor* females was much longer than that of males in the range of 15 °C to 25 °C.

### 3.2. The Effect of Different Temperatures on the Oviposition and Adult Emergence of H. hebetor

In general, the oviposition duration of *H. hebetor* decreased with increasing temperature, which reached the maximum of 50.78 d at 15 °C, and the minimum of 10.67 d at 35 °C (Table 2). The total number of eggs laid, the number of emerged adults, the emergence ratio, and the percentage of female offspring increased with increasing temperature in the range of 15 °C to 30 °C. 

### 3.3. Temperature-Dependent Survival of H. hebetor

Overall, the survival rates of different life stages of *H. hebetor* were as follows at the same temperature: pupae ≥ larvae > eggs (Table 3). The egg hatchability increased with increasing temperature in the range of 15 °C to 30 °C and decreased at 35 °C, which reached the maximum of 83.89% at 30 °C. The survival rate of *H. hebetor* larvae reached the minimum of 58.22% at 15 °C, and there was no significant difference at 20, 25, 30, and 35 °C (*F* = 4.42; *df* = 4.70; *p* > 0.05). There was also no significant difference in the survival rate of pupae of *H. hebetor* in the range of 15 °C to 35 °C (*F* = 0.73; *df* = 4.70; *p* > 0.05). 

### 3.4. Temperature-Dependent Development Times of H. hebetor

The developmental duration of *H. hebetor* decreased with increasing temperature from 15 °C to 35 °C (Table 4). The longest developmental duration of egg-to-adult stage was 48.46 d at 15 °C, and the shortest was 9.80 d at 35 °C. In the range of 15 °C to 35 °C, the developmental duration of *H. hebetor* pupae was the longest, reaching 30.58 d at 15 °C.

### 3.5. Developmental Threshold Temperatures and Effective Accumulated Temperatures of H. hebetor

The developmental threshold temperatures, effective accumulated temperatures, and predictive equations of the developmental duration of *H. hebetor* eggs, larvae, pupae, pre-oviposition stage, and egg-to-adult stages in the range of 15 °C to 35 °C are listed in Table 5. There was a positive correlation between ambient temperature and development rate for different life stages of *H. hebetor*.

The developmental threshold temperature of the whole generation (egg-to-adult stage) of *H. hebetor* was 9.29 °C, and the developmental threshold temperatures of different life stages of *H. hebetor* were listed in ascending order as follows: 6.39 °C for larvae, 9.24 °C for pupae, 13.53 °C for pre-oviposition stage, and 13.89 °C for eggs, respectively. The effective accumulated temperature of the whole generation (egg-to-adult stage) of *H. hebetor* was 240.31 °C·d.

### 3.6. Temperature-Dependent Population Dynamics of H. hebetor

The net productive rate, innate rate of increase, and population doubling time of *H. hebetor* are listed in Table 6. The innate rate of increase increased with increasing temperature, reaching the maximum of 0.19 at 30 °C and 35 °C. The highest net reproductive rate was 99.49 at 30 °C. The maximum population doubling time was 69.92 d at 15 °C.

## 4. Discussion

The present results showed that *H. hebetor* could complete development from egg to adult on *E. elutella* larvae in the temperature range of 15 °C to 35 °C, and the generation duration of *H. hebetor* gradually decreased with increasing temperature. The emergence rate and life span of *H. hebetor* was similar to Zeng et al. and Tian et al. [14,15].

The total number of eggs laid by *H. hebetor* reached the maximum of 192.39 at 30 °C. This was also similar to previous research results [12,16,17], where *G. mellonella* larvae and *P. interpunctella* larvae were used as hosts. However, the maximum emergence rate of *H. hebetor* reached 70.08% when *E. elutella* larvae were used as hosts in the present study, which was significantly higher than (35.87%) when *G. mellonella* larvae were used as hosts in Tian et al. [14]. When *Cadra cautella* larvae were used as hosts at 27 ± 2 °C, the egg hatchability, pupation rate, and adult emergence rate were more than 95% [18].

Net reproductive rate refers to the ability of female individuals in a population to give birth to the total number of new female individuals within a given time. Intrinsic increase rate refers to the maximum increase capacity (*r_m_*) of a population that can be observed under unrestricted conditions, such as being able to eliminate adverse weather conditions, provide ideal food conditions, and eliminate predators and diseases. Both can reflect the increased potential of a population. The net reproductive rate and intrinsic increase rate of *H. hebetor* reached the maximum at 30 and 35 °C, which is consistent with Golizadeh et al. [19] who reared *H. hebetor* on *A. kuehniella* larvae. The optimum temperature for the growth and development of *H. hebetor* is 30 °C [14,19]. Taking into account factors such as the total number of eggs laid, egg hatchability, number of emerged adults, emergence ratio, percentage of female offspring, innate rate of increase, and net reproductive rate, it is believed that optimal temperature for rearing of *H. hebetor* on *E. elutella* larvae is 30 °C in the current study.

This current study was carried out at constant temperatures (15, 20, 25, 30, and 35 °C), 75 ± 5% RH, and a photoperiod of 12:12 h (L:D), and the life parameters of *H. hebetor* under ambient conditions require further investigation, which is more in line with the actual application scenario of biological control. In addition, the present results provide more choices for determining suitable hosts for *H. hebetor* mass rearing. Especially, *E. elutella* is the most destructive Lepidopterous pest insect in tobacco storage spaces, and it is very interesting to investigate if *H. hebetor* reared on *E. elutella* larvae has stronger potential in controlling *E. elutella* infestation.

In addition, integrated pest management (IPM) has become the main direction of pest control. IPM emphasizes the coordinated application of multiple pest control methods [20,21,22]. Combining the application of *H. hebetor* and *Metarhizium anisopliae* (Metsch.) is compatible for effectively controlling *Ephestia kuehniella* Zeller larvae [23,24]. The combined application of *Heterorhabditis indica* (Rhabditida: Heterorhabditidae) and *H. hebetor* may be beneficial for the control of *P. interpunctella* [25]. Carifend^®^, an alpha-cypermethrin-coated polyester net, provides a satisfactory level of protection to stored tobacco against *Ephestia elutella* [26]. *Ephestia kuehniella* exhibits different behavioral preferences towards surfaces treated with diatomaceous earth (DE) and spinosad formulations [27]. Thus, whether the combination of *H. hebetor* and other control measures, such as physical control, chemical control, or other biological control methods, can effectively control *E. elutella* should be investigated in the future.

## 5. Conclusions

In conclusion, *H. hebetor* could complete growth and development when *E. elutella* larvae were used as hosts, and the developmental duration decreased with increasing temperature at 15, 20, 25, 30, and 35 °C. The development threshold temperatures of *H. hebetor* eggs, larvae, pupae, and egg-to-adult stages were 13.89, 6.39, 9.24, and 9.29 °C, and the effective accumulated temperatures were 23.33, 46.40, 142.68, and 240.31 °C·d, respectively. The total number of eggs laid by *H. hebetor*, the hatching rate of *H. hebetor* eggs, and the percentage of female offspring reached the maximum of 192.39, 83.89%, and 74.04% at 30 °C, respectively. There was no significant difference in pupal survival rate in the temperature range of 15 °C to 35 °C. At 30 °C, the pre-oviposition duration of *H. hebetor* was the shortest (0.87 d). The innate rate of increase and net reproductive rate reached the maximum at 30 °C. Therefore, the optimal rearing temperature of *H. hebetor* was 30 °C. The present results are in favor of the large-scale rearing of *H. hebetor* using *E. elutella* larvae as hosts for controlling Lepidopterous pests in practice.

## Figures and Tables

**Table 1:** The longevity of *Habrobracon hebetor* adults at 15, 20, 25, 30, and 35 °C, at 75 ± 5% RH, respectively.

Temperature (°C)	The Longevity of *H. hebetor* Adults (d)	
	Female	Male
15	51.41 ± 0.75 aA	11.09 ± 0.09 aB
20	29.35 ± 0.36 bA	12.15 ± 1.06 aB
25	13.76 ± 0.99 cA	7.03 ± 0.11 bB
30	9.81 ± 0.34 dA	6.26 ± 0.37 bA
35	6.69 ± 0.26 eA	3.09 ± 0.11 cA

Note: Data in the table are means and standard errors. Temperature and sex were used as two independent variables, and the adult longevity was used as a response variable for two-way analysis of variance (ANOVA). Within the same column, means followed by different lower letters indicate significant difference according to Tukey’s test (*p* < 0.05). Within the same row, means followed by different capital letters indicate significant difference according to *T*-test (*p* < 0.05).

**Table 2:** The effect of different temperatures on the oviposition and adult emergence of *Habrobracon hebetor*.

Temperature (°C)	Total Oviposition Duration (d)	The Total Number of Eggs Laid	The Number of Emerged Adults	Emergence Ratio (%)	Percentage of Female Offspring (%)
15	50.78 ± 0.86 a	112.12 ± 2.26 b	6.09 ± 0.13 d	5.44 ± 0.21 d	43.92 ± 3.86 d
20	22.08 ± 0.97 b	131.00 ± 2.08 b	22.32 ± 2.06 d	17.08 ± 1.73 d	50.28 ± 0.25 c
25	11.97 ± 0.37 cd	167.43 ± 24.01 ab	60.30 ± 10.79 c	35.60 ± 1.74 c	64.78 ± 0.83 b
30	12.91 ± 0.37 c	192.39 ± 6.89 a	134.51 ± 2.74 a	70.08 ± 2.71 a	74.04 ± 0.15 a
35	10.67 ± 0.51 d	163.65 ± 27.50 ab	80.10 ± 4.60 b	51.86 ± 8.78 b	54.77 ± 0.41 c

Note: Data in the table are means and standard errors. Within the same column, means followed by different lower letters indicate significant difference according to Tukey’s test (*p* < 0.05).

**Table 3:** Egg hatchability and survival rate of larvae and pupae of *Habrobracon hebetor* reared on *Ephestia elutella* larvae.

Temperature (°C)	Egg Hatchability (%)	Survival Rate of Larvae (%)	Survival Rate of Pupae (%)
15	26.43 ± 2.38 dC	58.22 ± 5.12 bB	87.54 ± 6.73 A
20	55.49 ± 2.79 cB	84.46 ± 3.77 aA	89.40 ± 2.47 A
25	57.89 ± 3.30 bcB	82.77 ± 7.74 aA	94.60 ± 2.72 A
30	83.89 ± 5.97 aA	87.87 ± 7.74 aA	94.12 ± 1.92 A
35	67.87 ± 0.93 bB	89.84 ± 5.12 aA	91.71 ± 1.25A

Note: Data in the table are means and standard errors. Within the same column, means followed by different lower letters indicate significant difference according to Tukey’s test (*p* < 0.05). Where no letters exist, no significant differences were noted.

**Table 4:** Developmental duration of *Habrobracon hebetor* reared on *Ephestia* larvae at 15, 20, 25, 30, and 35 °C, 75 ± 5% RH, respectively.

Temperature (°C)	Egg (d)	Larva (d)	Pupa (d)	Pre-Oviposition Stage (d)	Egg-to-Adult Stage (d)
15	8.67 ± 0.13 aC	5.30 ± 0.09 aC	30.58 ± 0.49 aB	3.91 ± 0.13 aC	48.46 ± 0.65 aA
20	3.86 ± 0.02 bC	3.43 ± 0.25 bC	12.43 ± 0.10 bB	2.44 ± 0.07 bC	22.16 ± 0.36 bA
25	2.09 ± 0.06 cC	2.26 ± 0.08 cC	7.86 ± 0.04 cB	1.09 ± 0.05 cdC	13.29 ± 0.06 cA
30	1.80 ± 0.09 dC	2.18 ± 0.08 cC	7.08 ± 0.11 dB	0.87 ± 0.03 dC	11.94 ± 0.02 dA
35	1.03 ± 0.10 eC	1.60 ± 0.05 dC	5.86 ± 0.05 eB	1.31 ± 0.09 cC	9.80 ± 0.06 eA

Note: Data in the table are means and standard errors. Within the same column, means followed by different lower letters indicate significant difference according to Tukey’s test (*p* < 0.05). Within the same row, means followed by different capital letters indicate significant difference according to Tukey’s test (*p* < 0.05). Each treatment started from 30 eggs of *H. hebetor* in the experiment.

**Table 5:** Linear regression equations, developmental threshold temperatures, and effective accumulated temperatures of different life stages of *Habrobracon hebetor* calculated according to the observation data at 15, 20, 25, 30, and 35 °C, at 75 ± 5% RH.

Developmental Stage	Linear Regression Equation	R^2^	Development Threshold Temperature (°C)	Effective Accumulated Temperature (°C·d)	Predictive Equation of the Development Duration
Egg	T = 23.335 V + 13.889	0.97	13.89 ± 2.20	23.33 ± 3.93	N = (23.33 ± 3.93)/T − (13.89 ± 2.20)
Larva	T = 46.405 V + 6.391	0.98	6.39 ± 3.31	46.40 ± 7.73	N = (46.40 ± 7.73)/T − (6.39 ± 3.31)
Pupa	T = 142.677 V + 9.242	0.98	9.24 ± 2.87	142.68 ± 23.77	N = (142.68 ± 23.77)/T − (9.24 ± 2.87)
Pre-oviposition stage	T = 16.406 V + 13.530	0.76	13.53 ± 2.73	16.41 ± 3.53	N = (16.41 ± 3.53)/T − (13.53 ± 2.73)
Egg-to-adult stage	T = 240.313 V + 9.293	0.98	9.29 ± 2.85	240.31 ± 39.88	N = (240.31 ± 39.88)/T − (9.29 ± 2.85)

Note: “T” stands for temperatures (°C), “N” stands for the developmental duration (d), and “V” stands for developmental rates (V = 1/N).

**Table 6:** Innate rate of increase and population doubling time of *Habrobracon hebetor* reared on *Ephestia elutella* larvae.

Temperature (°C)	Net Productive Rate (Times)	Innate Rate of Increase (d^−1^)	Population Doubling Time (d)
15	2.67 ± 0.22 c	0.01 ± 0.00 d	69.92 ± 6.13 a
20	11.21 ± 0.71 c	0.05 ± 0.00 c	12.82 ± 0.67 b
25	39.92 ± 0.11 b	0.14 ± 0.01 b	5.12 ± 0.26 b
30	99.49 ± 0.05 a	0.19 ± 0.00 a	3.60 ± 0.02 b
35	43.83 ± 0.13 b	0.19 ± 0.00 a	3.60 ± 0.70 b

Note: The net reproductive rate of insects refers to the multiplication rate of a population after one generation under certain conditions. Data in the table are means and standard errors. Within the same column, means followed by different lower letters indicate significant difference according to Tukey’s test (*p* < 0.05).

## Data Availability

Data are available upon request.
